# The Impact of Covariates in Voxel-Wise Lesion-Symptom Mapping

**DOI:** 10.3389/fneur.2020.00854

**Published:** 2020-08-14

**Authors:** Deepthi Rajashekar, Matthias Wilms, Kent G. Hecker, Michael D. Hill, Sean Dukelow, Jens Fiehler, Nils D. Forkert

**Affiliations:** ^1^Department of Radiology, University of Calgary, Calgary, AB, Canada; ^2^Hotchkiss Brain Institute, University of Calgary, Calgary, AB, Canada; ^3^Departments of Community Health Sciences and Veterinary Clinical, and Diagnostic Sciences, University of Calgary, Calgary, AB, Canada; ^4^Department of Clinical Neurosciences, University of Calgary, Calgary, AB, Canada; ^5^Department of Diagnostic and Interventional Neuroradiology, University Medical Center Hamburg-Eppendorf, Hamburg, Germany; ^6^Alberta Children's Hospital Research Institute, University of Calgary, Calgary, AB, Canada

**Keywords:** brain, lesion symptom mapping, analysis of variance, general linear model, stroke

## Abstract

**Background:** Voxel-wise lesion-symptom mapping (VLSM) is a statistical technique to infer the structure-function relationship in patients with cerebral strokes. Previous VLSM research suggests that it is important to adjust for various confounders such as lesion size to minimize the inflation of true effects. The aim of this work is to investigate the regional impact of covariates on true effects in VLSM.

**Methods:** A total of 222 follow-up datasets of acute ischemic stroke patients with known NIH Stroke Scale (NIHSS) score at 48-h post-stroke were available for this study. Patient age, lesion volume, and follow-up imaging time were tested for multicollinearity using variance inflation factor analysis and used as covariates in VLSM analyses. Covariate importance maps were computed from the VLSM results by standardizing the beta coefficients of general linear models.

**Results:** Covariates were found to have distinct regional importance with respect to lesion eloquence in the brain. Age has a relatively higher importance in the superior temporal gyrus, inferior parietal lobule, and in the pre- and post-central gyri. Volume explains more variability in the opercular area of the insula, inferior frontal gyrus, and caudate. Follow-up imaging time accounts for most of the variance in the globus pallidus, ventromedial- and dorsolateral putamen, dorsal caudate, pre-motor thalamus, and the dorsal insula.

**Conclusions:** This is the first study investigating and revealing distinctive regional patterns of importance for covariates typically used in VLSM. These covariate importance maps can improve our understanding of the lesion-deficit relationships in patients and could prove valuable for patient-specific treatment and rehabilitation planning.

## Introduction

Voxel-wise lesion symptom mapping (VLSM) is a statistical framework that can be used to quantify the regional relationship of structural integrity of the brain (post-stroke) to a clinical outcome of interest. In the context of acute ischemic strokes, previous literature has investigated these lesion-deficit relationships at the regional or voxel level using various measures of stroke severity of varying granularity ranging from gross outcomes, like the modified Rankin scale (1, 2), to finer measures of impairment, for example to assess language (3, 4), spatial neglect (5), and proprioception (6). The results from VLSM are population-specific observations that can provide new insights into mechanisms underlying stroke recovery and, therefore, have potential to guide future research in stroke precision medicine.

Various factors such as lesion size, lesion location (7), age (8), sex (9), time to treatment (10, 11), blood pressure (12), and prevailing medical conditions (13) of the patient have been previously identified to be important parameters for stroke treatment decision making. The effects of these (and other) confounding variables might be related to either the extent of structural damage or the severity of clinical outcome (the relationship studied in the VLSM analyses). This suggests that VLSM analyses should take these confounders into account to produce maps of the true regional eloquence, i.e., the underlying structure-function relationship that indicates the brain regions that are highly critical (eloquent) with respect to the clinical outcome of interest. This can, for example, be practically implemented in voxel-level methods by including the confounders as covariates in a regression model. To date, the VLSM literature has dominantly considered age, sex, and lesion volume as covariates with relevance to stroke (14).

However, to the best of our knowledge, there is no work that quantifies the relative importance each covariate has on the voxel-level statistic of the VLSM output. Therefore, the aim of this work is to estimate the importance of each covariate at a voxel level using a VLSM technique. The proposed covariate importance maps add complementary information to the standard VLSM output, which could be a valuable tool for acute treatment decision making as well as tailored rehabilitation planning.

## Methods

### Datasets

The datasets available and used for this study are obtained from the two multi-center ESCAPE (15) and IKNOW (2) trials, which enrolled patients with middle cerebral artery stroke (MCA). In this work, patients with severe white matter hyperintensities, bilateral strokes, and remote hemorrhagic transformations are excluded. Patients who obtained a follow-up FLAIR MRI or non-contrast CT imaging (18 hours−7 days from baseline) and had a complete clinical assessment within 48-hours of symptom onset are included in this study. The final sample contains 222 subjects (98 women) with an average age of 68.6 ± 12.6 years. The clinical outcome of interest used in this work is the NIH Stroke Scale (NIHSS) assessed at 48-hours post-stroke. NIHSS is a commonly used secondary stroke outcome score involving assessments for (in decreasing order of representation) voluntary motor function, level of consciousness, vision, language, sensory function, and spatial neglect. The 48-hours timepoint is selected to avoid biases in the results due to comorbidities unrelated to stroke and complications arising from in-hospital treatment at later assessment timepoints. All datasets used in this study were made available for this secondary study after complete anonymization.

### Pre-processing

All lesions are segmented by an experienced observer using ITK-SNAP (16). After this, all datasets are skull-stripped and registered non-linearly to a common FLAIR and NCCT atlas (17) using cost function masking, implemented in the ANTs toolkit (18). Subsequently, the computed deformation field that maps the native patient scan to the atlas image is applied to the corresponding binary lesion mask for that patient. Since the dataset is pooled from multicenter trials, there is considerable variability in: in-plane resolution [0.37–1.4 mm^2^], slice thickness [2–10 mm], and the number of slices acquired [4–87]. Registering all native patient scans to a common atlas not only helps to minimize image acquisition related differences but also removes anatomical differences between all patients and allows for an unbiased statistical analysis within the common atlas space.

All datasets were visually inspected to ensure that no motion or other imaging artifacts are present, signal to noise ratio was suitable, and the acquisitions were complete covering the whole brain. Likewise, the registration results were visually checked and datasets with sub-optimal registration quality were excluded from this LSM analysis.

### Voxel-Wise Lesion Symptom Mapping

Voxel-wise lesion symptom mapping (VLSM) is a statistical technique to generate eloquence maps that quantify the difference between patients with a lesion and those without a lesion in each voxel (3) with respect to a clinical outcome score. The result of VLSM is a parametric map that displays the eloquence of each voxel with respect to the clinical outcome score of interest, known as the lesion-symptom map.

Practically, this can be implemented by a voxel-wise statistical test comparing the distributions of the outcome scores in patients with a lesion in a voxel to patients without a lesion in the same voxel. This procedure results in a t-score, indicating how critical that voxel is with respect to the outcome score of interest. Voxels with higher t-scores are deemed to be more eloquent (i.e. critically associated) to the outcome score (here the 48-hours NIHSS), thereby quantifying the structure-function relationship. In other words, a high average t-score within a brain region implies that a lesion in this brain region likely leads to more severe clinical deficits. In this work, each voxel is modeled as a general linear model (GLM) for VLSM, which is one of the traditional methods to quantify lesion-deficit relationships (3). Correction for multiple comparisons was done using the permutation based thresholding approach (19). The proposed framework for variance estimation is an extension of the VLSM source code released by Bates et al. (3).

### Variable Importance

In this work, patient age, lesion volume, and the time from symptom onset to follow-up imaging are selected as covariates to explore regional covariate importance.

These covariates are tested for collinearity in a first step using the Spearman's correlation, which is further confirmed by a variance inflation factor analysis (20) using a linear regression model to predict NIHSS. Once ensured that the covariates are unrelated, the VLSM analyses involved modeling the independent voxel-wise GLMs (21) as shown below and correcting for multiple comparisons.

(1) $$\begin{array}{l} S_{i}={\beta_{i,1}*L}_{i}+\beta_{i,2}*age+\beta_{i,3}*vol+\beta_{i,4}*fup+\epsilon_{i} \end{array}$$

Here, for each voxel location *i*, an independent regression model that predicts the eloquence score *S*_*i*_ at that spatial location using presence of lesion at that location (*L*_*i*_*)*, age, lesion volume, and follow-up imaging time (fup) as inputs is estimated. However, only voxels that survived the permutation threshold (maximum t-threshold = 5.06 from the non-parametric null max distribution over 1,000 permutations at alpha = 0.05) are considered to remove false positives.

The relative importance of covariates is inferred from the standardized beta coefficients for each covariate *j* at the voxel location *i* (β*s*_*i,j*_). Practically, the non-standardized beta coefficient of a covariate (β_*i,j*_) is normalized by the variance of the covariate and the mean squared error at the voxel location *i* as follows (22).

(2) $$\begin{array}{l} \beta s_{i,j}=\frac{\beta_{i,j}}{\sqrt{var\left(\beta_{i,j}\right)}*MSE_{i}} \end{array}$$

Subsequently, the standardized beta coefficients for a covariate at each true positive eloquent voxel is written out into a separate covariate importance map, resulting in age-, volume-, and follow-up time-specific covariate importance maps. These individual covariate importance maps are linearly normalized to the range 0–1 to enable comparison.

For ease of interpretation, the parcellation defined in the Brainnetome atlas (23) is used to calculate average regional covariate importance estimates. Finally, hierarchical clustering is employed to group brain regions based on the average region-level importance measures for each covariate. An important advantage of using hierarchical clustering is that, unlike flat clustering techniques, it provides a structural grouping of cerebral subregions. In order to avoid isolated eloquent regions with few voxels biasing the clustering algorithm, an overlap analysis is conducted. More precisely, all brain regions as defined by the Brainnetome atlas are ordered by the volume of overlap with the VLSM output and only those regions that have at least a volume overlap of 400 voxels (50th percentile) are included in the clustering analysis.

## Results

**Figure 1:**
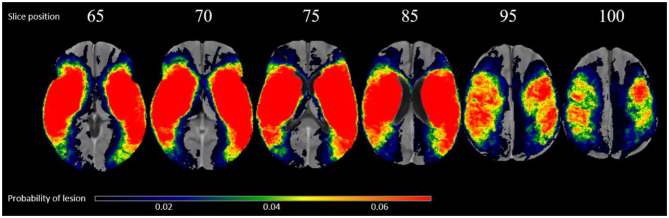
Overlap of all patients lesions in the common atlas space (*N* = 222) in radiological convention.

Of the 222 subjects, there are 100 right hemispheric strokes. The average volume of lesions on the left and right hemisphere was 42.04 and 43.65 cm^3^, respectively. The overlap of all transformed lesions on the common FLAIR and NCCT atlas shows a typical distribution for MCA occlusions, shown in Figure 1.

The correlation coefficients (r_s_) comparing all covariates at the patient level are <0.1 (*p* > 0.05) suggesting that there is no monotonic association between any two covariates (r_s_ values: age vs. lesion volume: −0.086; age vs. follow-up time: −0.051; volume vs. follow-up time: 0.043). This finding is further confirmed by their variance inflation values being <5.0 in a linear regression model to predict the 48-hours NIHSS, which is typically considered to indicate the absence of multicollinearity in the input data (24).

**Figure 2:**
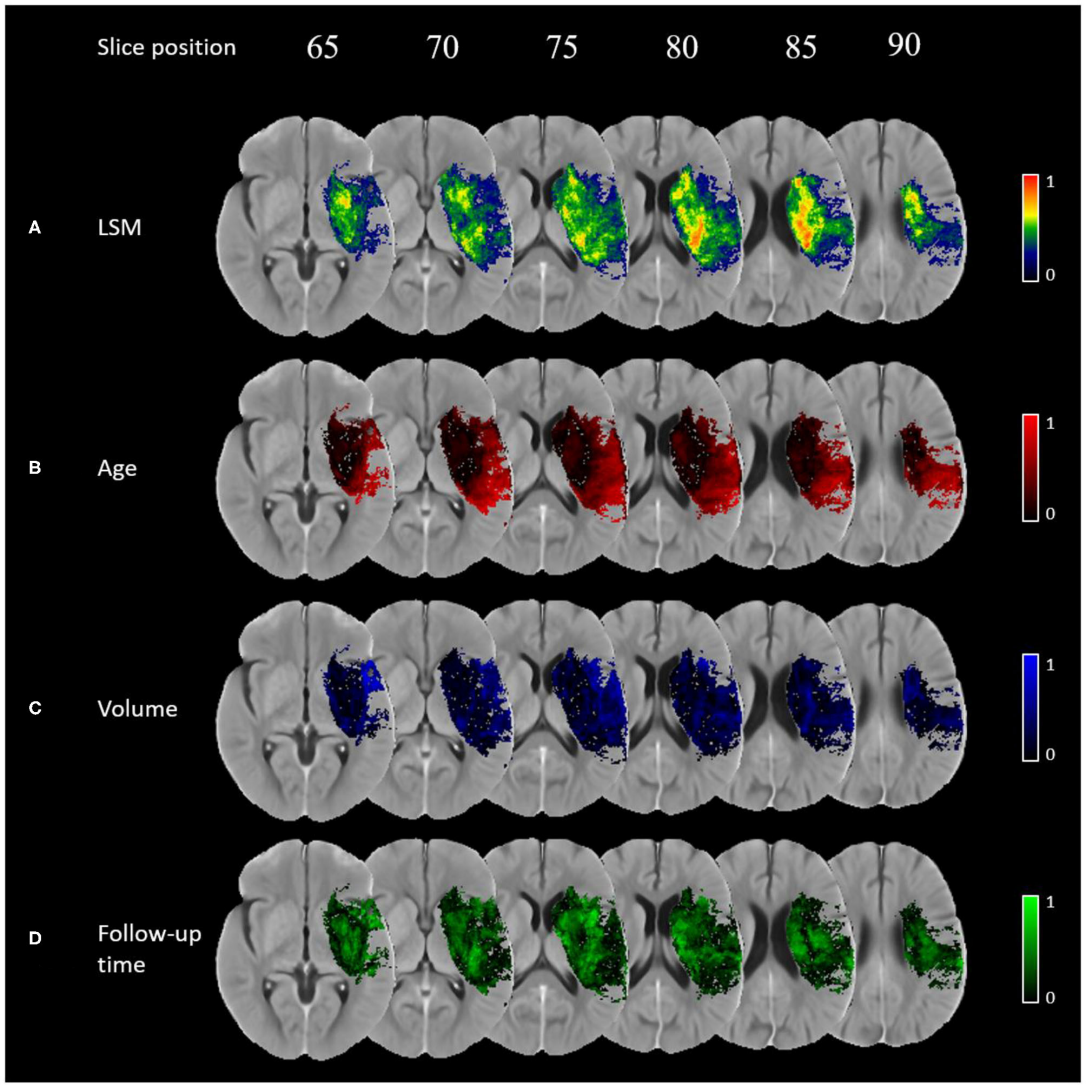
Importance maps for each covariate using voxel-wise generalized linear models corrected for multiple corrections using permutation-based thresholding (*p*-value < 0.05): **(A)** normalised VLSM result, **(B)** age, **(C)** lesion volume, **(D)** follow-up imaging time.

The normalized VLSM output is shown in Figure 2A. Brain regions with relatively higher t-score values are considered more eloquent with respect to the NIHSS outcome scale, which means that even a small lesion volume in these regions is likely to result in a worse outcome. In this work, the eloquent clusters that survived the correction for multiple comparisons are located around the sub-cortical left hemispheric regions. No eloquence in the right hemisphere is observed.

The importance maps are substantially different for the covariates investigated in this work (see Figures 2B–D). Age has a relatively higher importance in the superior temporal gyrus, inferior parietal lobule, and in the pre- and post-central gyri. Lesion volume has the highest relative importance in the opercular area of the inferior frontal gyrus, ventral caudate, and the ventral agranular insula. Finally, follow-up time was found to be the most important covariate in the globus pallidus, ventromedial- and dorsolateral putamen, dorsal caudate, pre-motor thalamus, and dorsal insula.

**Figure 3:**
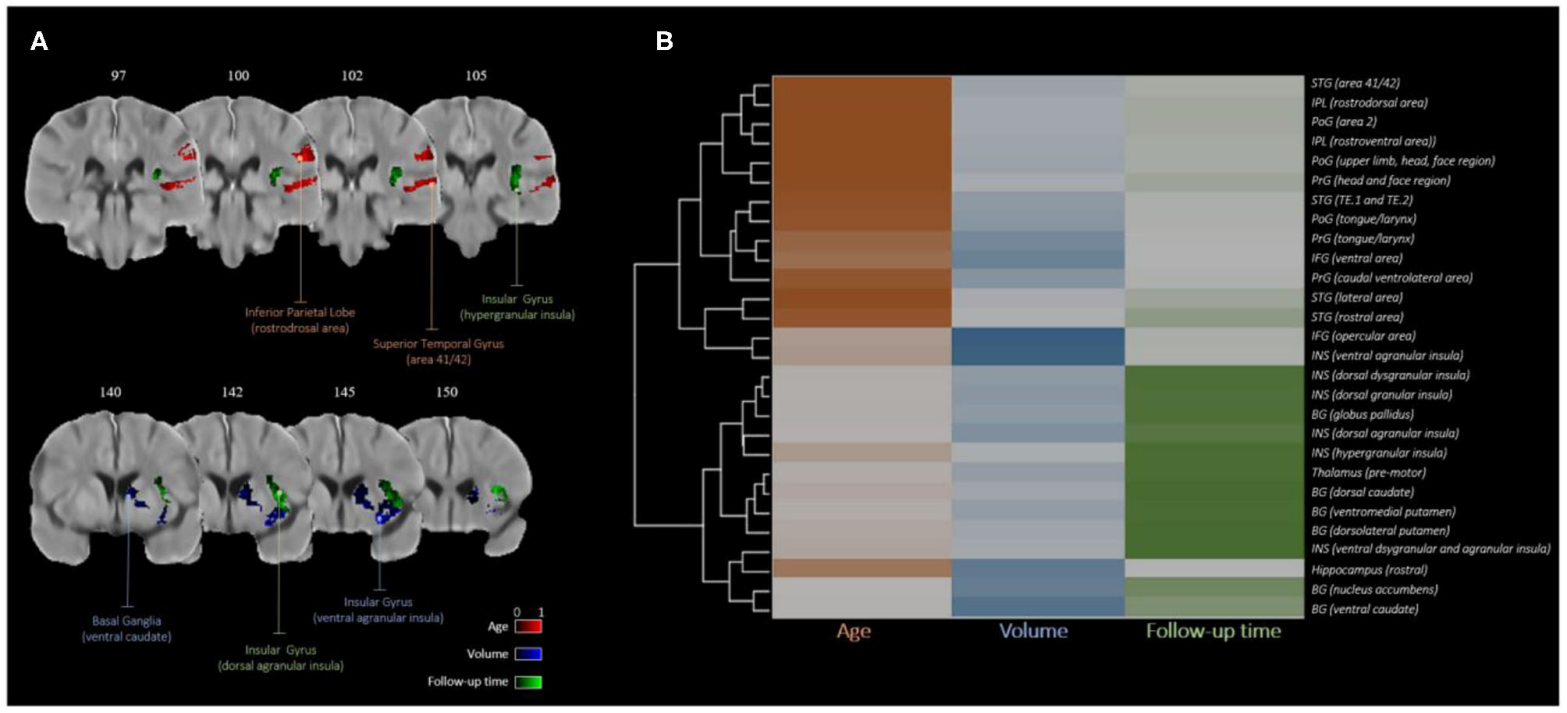
Covariate importance estimates consolidated by hierarchical clustering: **(A)** regions with higher average importance for age (red), volume (blue), and follow-up time (green); **(B)** dendrogram of brain regions clustered by relative covariate importance.

The result from the hierarchical clustering algorithm is shown in Figure 3A. Here, the heatmap from the clustering algorithm is represented as a dendrogram in Figure 3B outlining the sub-regions with considerable overlap with the VLSM map. In the heatmap, each covariate is color-coded (age in orange, lesion volume in blue, and follow-up time in green) with darker hues representing higher average covariate importance for a given brain region. It is clear from the heatmap that for each covariate, the set of brain regions with high relative importance is nearly exclusive.

## Discussion

The main finding of this study is that covariates typically used within VLSM analyses show distinctive patterns of regional importance for modeling lesion eloquence.

Of the eloquent brain regions mainly influenced by age, the superior temporal gyrus and inferior parietal lobule have been previously reported to display age-specific changes in cerebral blood flow (CBF) patterns in healthy elderly (61.05 ± 13.17 years of age) (25). Specifically, it was previously reported that the CBF in the superior temporal gyrus increases with age as a compensatory response to cognitive tasks, i.e., increased neural activity. Contrary to this, the CBF in the inferior parietal lobule was shown to have a negative correlation with age resulting from the reduction in neuronal activity and deterioration of microvasculature. Other studies using various imaging modalities to investigate age-related perfusion changes have also led to comparable conclusions in these regions (26, 27). The age-related changes in CBF, microvasculature, and neuronal and synaptic activity may deem these cortical structures more susceptible to brain damage in elderly including ischemic stroke, thereby also explaining the likelihood of superior temporal gyrus and inferior parietal lobule not only being eloquent to stroke severity metrics, but also their structure-function relationship most explained by age.

From a connectivity perspective, the hippocampus, basal ganglia structures, and insula are highly connected structures (*a.k.a.*, rich-club structures) (28), indicating that any insult to these regions is likely to result in a poor clinical outcome. This supports the current finding that lesion volume is the most important covariate for these rich-club brain structures or structures that link to a rich club node, such as the caudate and insula (29).

The brain regions that have been previously reported to have the highest ischemic vulnerability (i.e., increase in infarct per unit reduction in CBF) are the caudate body, putamen nucleus, insular ribbon, middle frontal gyrus, precentral gyrus, and the frontal lobe subcortical white-matter and paracentral lobule (30). Furthermore, the insular ribbon has been described as the most vulnerable brain region of the left hemisphere (30). While it is important to include follow-up imaging time as a covariate to account for potential lesion growth/shrinkage, secondary injuries, and water accumulation differences over time, the importance of follow-up imaging time (or the post-treatment scan time) in the insular gyrus specifically, remains unclear, requiring further research. In general, the regions that are common in variance importance maps have an overall high eloquence, i.e., critical to the outcome of interest. From Figure 3B, it is clear that even though there are common regions in the importance maps for each covariate, there are differences in average importance across regions, suggesting that one covariate is likely to be relatively more important than the other two covariates.

There is a strong evidence in the stroke literature pertaining to the bias of the NIHSS assessment. More precisely, the NIHSS is biased toward the left hemisphere because of the language domains and the fact that the consciousness domains are weighted to language. The right hemisphere is reported to have a relatively less weight in NIHSS. Particularly, it was shown that the volume of a right hemispheric lesion has to be far greater than a left hemispheric insult to result in the same severity of outcome on the NIHSS scale (31). A recent VLSM analysis conducted on 216 subjects from the MR CLEAN study showed that the inclusion of lesion volume as a covariate eliminates the eloquence signal in the right hemisphere (1). They described the resulting LSM maps from three scenarios: (1) using the outcome score alone, (2) using the outcome score as the target variable and sex and age as covariates; and (3) using the outcome score as the target variable and sex, age, and lesion volume as covariates [see Figure 2 in Ernst et al. (1)]. The results clearly indicate that the right hemispheric eloquence is no longer present when lesion volume is added as a covariate in the LSM analysis using the modified Rankin score as the outcome score. Therefore, the absence of eloquence in the right hemisphere in this study is likely to stem from either the lateralization of the NIHSS assessment, the effects of lesion volume as a covariate, or both.

The limitations of the proposed method to estimate variance importance of covariates in a lesion symptom mapping approach should be discussed. First, this work should be considered exploratory in terms of the choice of covariates to be adjusted for or included in the analysis. Although the covariates selected in this work are motivated by previous clinical stroke literature and are typically considered in LSM analyses, this selection does not cover the entire repertoire of confounders that could potentially bias an LSM study. For example, sex was intentionally excluded from this study since there are established sex-specific associations with other clinical and lifestyle behaviors (9). Nevertheless, the results of this study suggest that it is of clinical interest to investigate the regional importance of covariates, and more co-variates should be investigated in future studies to improve our understanding of the structure-function relationship. Furthermore, the current implementation does not account for potential interaction terms. However, the proposed method could be extended to include interaction effects between covariates as part of the GLM as an additional regression term as follows.

(3) $$\begin{array}{l} S_{i}={\beta_{i,1}*L}_{i}+\beta_{i,2}*age+\beta_{i,3}*vol+\beta_{i,4}*fup+\\ \beta_{i,5}*\left(age*vol\right)\;+\;\beta_{i,5}*\left(vol*fup\right)+\epsilon_{i} \end{array}$$

However, interpretation of interaction effects is often complicated and requires that VLSM literature accumulates sufficient evidence of the independent effects of clinically relevant covariates.

Furthermore, voxel-wise LSM requires multiple comparisons correction leading to a low statistical power (32, 33) while not accounting for similar functional deficits induced by non-overlapping lesions (i.e., the partial injury problem) (34). While LSM research is leading toward multivariate models (35, 36) to resolve these issues, the general understanding of the influence of covariates in defining the structure-function relationship in these multivariate models remains unclear. Finally, the results reported in this study may be population-specific. That is, a different sample size, unreliable segmentations of the lesions, or different lesion distributions could likely influence the VLSM analysis, and their impact cannot be easily quantified. Overall, this work should be considered as a first important step in the estimation of voxel-wise variance using the most traditional VLSM technique – general linear regression.

## Conclusions

To the best of our knowledge, this is the first study investigating the regional importance of covariates typically used in VLSM. Using the proposed method, distinctive patterns of regional importance of age, lesion volume, and follow-up time were found. The generated covariate importance maps can help to improve our understanding of the lesion-deficit relationships in patients and could prove valuable for patient-specific treatment and rehabilitation planning.

## Data Availability Statement

The data analyzed in this study is subject to the following licenses/restrictions: The acquisition of the data sets for the two trials was approved by the respective local ethics board at each site contributing to the two trials. All data sets used in this secondary study were made available after complete anonymization. Requests to access these datasets should be directed to Nils Forkert, nils.forkert@ucalgary.

## Ethics Statement

Ethical review and approval was not required for the study on human participants in accordance with the local legislation and institutional requirements, as all datasets were fully anonymized prior to transfer and processing. Our paper is a retrospective (secondary) study, not requiring review and approval.

## Author Contributions

DR and MW: study design, image processing, data analysis, drafting the manuscript, and revising it critically. NF, KH, and SD: drafting the manuscript and revising it critically. JF and MH: data acquisition and revising the manuscript critically. All authors contributed to the article and approved the submitted version.

## Conflict of Interest

The authors declare that the research was conducted in the absence of any commercial or financial relationships that could be construed as a potential conflict of interest.
